# High bicarbonate replacement fluid and time to pH normalization during continuous veno-venous hemofiltration with regional citrate anticoagulation: a retrospective single-center cohort study

**DOI:** 10.1093/ckj/sfaf117

**Published:** 2025-04-26

**Authors:** Timo Mayerhöfer, Paul Köglberger, Fabian Perschinka, Georg F Lehner, Lisa Schilchegger, Romuald Bellmann, Andreas Peer, Birgit Zassler, Sebastian Schauflinger, Michael Joannidis

**Affiliations:** Division of Intensive Care and Emergency Medicine, Department of Internal Medicine, Medical University Innsbruck, Innsbruck, Austria; Division of Intensive Care and Emergency Medicine, Department of Internal Medicine, Medical University Innsbruck, Innsbruck, Austria; Institute of Anesthesiology and Critical Care Medicine, Klinikum Wels, Wels, Austria; Division of Intensive Care and Emergency Medicine, Department of Internal Medicine, Medical University Innsbruck, Innsbruck, Austria; Division of Intensive Care and Emergency Medicine, Department of Internal Medicine, Medical University Innsbruck, Innsbruck, Austria; Division of Intensive Care and Emergency Medicine, Department of Internal Medicine, Medical University Innsbruck, Innsbruck, Austria; Division of Intensive Care and Emergency Medicine, Department of Internal Medicine, Medical University Innsbruck, Innsbruck, Austria; Division of Intensive Care and Emergency Medicine, Department of Internal Medicine, Medical University Innsbruck, Innsbruck, Austria; Division of Intensive Care and Emergency Medicine, Department of Internal Medicine, Medical University Innsbruck, Innsbruck, Austria; Division of Intensive Care and Emergency Medicine, Department of Internal Medicine, Medical University Innsbruck, Innsbruck, Austria; Division of Intensive Care and Emergency Medicine, Department of Internal Medicine, Medical University Innsbruck, Innsbruck, Austria

**Keywords:** acid-base disturbances, acidemia, critically ill, intensive care unit, renal replacement therapy

## Abstract

**Background:**

In critically ill patients, acid–base disorders are common before start of continuous renal replacement therapy. The aim of this study was to determine the influence of a high bicarbonate replacement fluid (30 mmol/L, Phoxilium^®^) on underlying acid–base disturbances.

**Methods:**

This single-center retrospective study included patients treated with continuous veno-venous hemofiltration (CVVH) at a medical ICU from January 2018 to May 2019. All patients received CVVH with regional citrate anticoagulation (RCA) and a high bicarbonate RF (Phoxilium^®^). Patients were categorized based on their initial pH. Acid–base parameters were closely monitored over 72 h at pre-specified intervals.

**Results:**

The study included 64 patients with a median age of 68 years. At the start of CVVH, 56.3% (*n* = 36) had acidemia, 12.5% (*n* = 8) had alkalemia and 32.3% (*n* = 20) had a normal pH. The median pH of patients with acidemia [0 h: 7.28 (interquartile range 7.23–7.33)] was corrected quickly to the normal range within 8 h [7.36 (interquartile range 7.29–7.4)]. The median pH of patients with alkalemia took longer (48 h) to reach normal values and patients with a normal pH showed a further pH increase within the normal range over the 72 h. All patients showed an increasing bicarbonate and base excess from 24 to 72 h.

**Conclusions:**

The RF in CVVH with RCA appears to be one of several factors influencing acid–base balance. Patients with different pre-existing acid–base disorders showed distinct correction kinetics. Prospective studies are needed to determine the clinical relevance of these findings and to optimize RF composition for better patient outcomes.

## Graphical Abstract

**Graphical Abstract:**
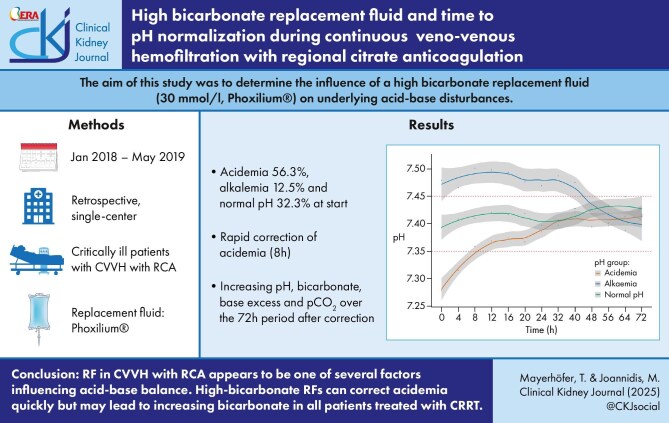

KEY LEARNINGS POINTS **What was known:**

- Acid–base disorders are common in critically ill patients requiring continuous renal replacement therapy (CRRT).
- High bicarbonate replacement fluids (RF) can potentially correct acidemia, but their wider effects on acid–base parameters over time are unknown.
- The clinical relevance of different bicarbonate RF remains unclear, and studies are needed to optimize RF selection.

**This study adds:**

- High bicarbonate RF (30 mmol/L) rapidly corrects acidemia in patients undergoing continuous veno-venous hemofiltration (CVVH) with regional citrate anticoagulation (RCA, 8 h).
- All groups show a further increase in bicarbonate and base excess after correction.
- High bicarbonate RFs may induce elevated bicarbonate levels, highlighting the need for an individualized approach to RF composition.

**Potential impact:**

- During CVVH with RCA with a high bicarbonate RF clinicians should closely monitor bicarbonate levels to avoid overcorrection.
- These findings support the need for further investigation into the complex influence of RF on acid–base disturbances in CVVH.

## INTRODUCTION

Acute kidney injury (AKI) is common in critically ill patients and is associated with increased mortality. Some patients require renal replacement therapy (RRT) to manage its complications [1]. In most intensive care units (ICU) RRT is delivered as intermittent haemodialysis or continuous renal replacement therapy (CRRT) [2].

Although no study has shown a survival benefit, CRRT does have some advantages, such as higher mean arterial pressure during therapy [3]. Therefore, continuous techniques are commonly used in critically ill, especially those with hemodynamic instability [4, 5].

Continuous techniques can be performed as convection (hemofiltration), diffusion (hemodialysis) or the combination of both (hemodiafiltration) [3]. Hemofiltration and hemodiafiltration techniques require a replacement fluid (RF) to replace the filtrate produced during the process. The evidence regarding the composition of these RFs is little and in the beginning of CRRT dialysates or dialysate-like fluids were used as RFs. Commercial RFs that are now available mainly attempt to mimic physiological electrolyte concentrations and require a buffer anion, as bicarbonate is lost during the filtration process (4). Consequently, the first available RFs contained relatively high concentrations of bicarbonate (5). In Europe, Phoxilium^®^ is a registered RF for continuous veno-venous haemofiltration (CVVH) and contains 30 mmol/L of bicarbonate. The use of bicarbonate in the critically ill patient with severe acidemia is controversial [6, 7]. For patients receiving CRRT a cohort study could even demonstrate an association between high bicarbonate concentration and mortality [8]. In addition, the increasing use of regional citrate anticoagulation (RCA) as recommended by the Kidney Disease: Improving Global Outcomes (KDIGO) guidelines [9], which involves the systemic metabolism of citrate to bicarbonate in the liver [10], reduces the necessity to deliver additional bicarbonate via the RF. Citrate is a weak acid with a minimal theoretical acidifying effect. However, when bound to ionized calcium, these complexes are rapidly metabolized and cleared from the bloodstream. According to the Stewart approach, blood pH is primarily influenced by three factors: PaCO₂, strong ion difference (SID) and weak acids. Some citrate solutions contain high sodium concentrations (with trisodium citrate) and impact acid–base balance by increasing the SID. As a result, the overall effect of citrate metabolism leads to plasma alkalinization [11].

Metabolic disorders in general are common before start of CRRT in the ICU. Acidemia is a frequent condition in critically ill patients with AKI [12]. However, there are also some patients who experience alkalemia before the start of RRT [13, 14]. The composition of RFs may interfere with acid–base parameters, particularly in patients with metabolic disorders.

Therefore, our aim was to investigate the trends of metabolic parameters in patients with acidemia, alkalemia, or a normal pH after the start of RRT for the first 72 h.

## MATERIALS AND METHODS

### Study design, setting and patients

This is a single-center retrospective study including patients treated with CVVH at the medical ICU in Innsbruck, who were admitted to ICU between January 2018 and April 2019. All patients were treated exclusively with CVVH using RCA during the study period. Management of RRT in our ICU has been described previously [15]. In short, RF was administered post-filter at a rate aimed to achieve 25 mL/kg/h, including pre-filter citrate solution. RCA was performed with a pre-filter infusion of Regiocit^®^ (prismocitrate 18/0, Gambro Lundia AB, Sweden). Calcium replacement consisted of a customized Ca^2+^ solution (calcium chloride 500 mmol/L, with or without magnesium chloride 250 mmol/L) infused post-filter via a separate central venous line. Table 1 shows the composition of the different solutions used in our CVVH circuit.

**Table 1:** Composition of solutions used during CVVH.

Solution	Na⁺ (mmol/L)	K⁺ (mmol/L)	Cl⁻ (mmol/L)	Ca²⁺ (mmol/L)	Mg²⁺ (mmol/L)	HCO₃⁻ (mmol/L)	Citrate (mmol/L)
Phoxilium^®^	140	4	115.9	1.25	0.6	30	0
Regiocit^®^ 18/0	140	0	86	0	0	0	18
Calcium solution	0	0	1000	500	0	0	0

HCO_3_^−^, bicarbonate.

Haemofiltration circuits were changed as needed according to clinical routine at latest after 72 h. CVVH was performed using the Baxter PrismafleX^®^ eXeed system according to the manufacturer's protocol and local standard operating procedures.

Inclusion criteria were as follows: individuals (i) aged 18 years or older, (ii) admitted to the ICU and (iii) deemed by the attending physician to require CVVH. Patients were only included while CVVH was performed using Phoxilium^®^.

Patients were excluded (i) if they had previously received RRT during the current hospitalization, (ii) if dialysis-dependent end-stage renal disease was present, or (iii) if there was incomplete documentation of CVVH data sheets. Patients (iv) with CVVH treatment duration less than 48 h and (v) if CVVH therapy was not started with Phoxilium were also excluded, to ensure a comparable exposure to the therapy (Supplementary data, Fig. S1).

The study was approved by the local ethic committee of the Medical University Innsbruck (Nr 1353/2022) and was performed in accordance with the Declaration of Helsinki and the European Data Policy.

### Definitions and data collection

Patient characteristics, clinical information and longitudinal laboratory parameters were extracted from the medical records, that were obtained during clinical routine. CVVH settings were obtained from CVVH data sheets completed during treatment. Acid–base parameters were extracted from the blood gas analysis at the beginning of the CVVH treatment and every 4 h during the first 24 h (0 h, 4 h, 8 h, 12 h, 16 h, 20 h, 24 h) and thereafter every 8 h (32 h, 40 h, 48 h, 56 h, 64 h, 72 h). Patients were followed for a maximum of 72 h, until an interruption of at least 12 h after reaching 48 h of CVVH, until RFs were switched to a low-bicarbonate solution, termination of CVVH or death, whichever occurred first. Patients were stratified according to the pH at the beginning of CVVH (normal pH: 7.35–7.45; acidemia: <7.35; alkalemia: >7.45). The primary outcome is the dynamic trend of pH during the 72 h of CVVH between the above-mentioned intervals. The SID was calculated to assess the acid–base balance based on the Stewart approach. SID represents the difference between strong cations and strong anions in plasma and was computed using the following equation:


\begin{eqnarray*}
SID = (N{a^ + } + {K^ + } + C{a^{2 + }} + M{g^{2 + }}) - (C{l^ - } + \textit{Lactat}{e^ - }).
\end{eqnarray*}

SID was calculated at specific time points (0 h, 24 h, 48 h, 72 h) for each patient.

### Statistical analysis

Categorical variables are presented as numbers with corresponding percentages and continuous variables are presented as median with interquartile range (IQR). Normal distribution of continuous data was checked with the Shapiro–Wilk test. Normally distributed data was compared using a two-sample *t*-test. Non-normally distributed data were compared using the Mann–Whitney U test, Kruskal–Wallis test or the χ^2^-test.

We calculated the median values of the the metabolic parameters for each timepoint and used a locally estimated scatterplot smoothing (LOESS) to smooth time trends of these values for each group (alkalotic, acidotic and normal pH). LOESS is a non-parametric regression method that models non-linear relationships by fitting multiple localized regressions across the data. The span (parameter alpha), which controls the degree of smoothing, was set at 0.75 and graphs are presented with 95% confidence intervals. Boxplots were used to visualize the distribution of key laboratory parameters across predefined timepoints (0 h, 24 h, 48 h, and 72 h). Box plots were used to visualize the distribution of the SID and other metabolic parameters at specific time points (0 h, 24 h, 48 h, 72 h). These plots display the median and IQR, and potential outliers.

A *P*-value <.05 was considered statistically significant and all statistical tests were two-sided. Statistical analysis was performed using R Software (4.4.0).

## RESULTS

### Patients

In total 64 patients were included in this analysis; a patient flowchart is provided in the Supplementary data, Fig. S1. Patient characteristics and laboratory parameters at initiation of RRT are presented in Tables 2a and 2b. Baseline characteristics stratified by the initial pH at the start of CVVH are provided in the Supplementary data, Tables S1a and S1b. The median age of the overall cohort was 68 years (IQR 57–76 years). Patients were predominantly male with 67.2% (*n* = 43). Chronic kidney disease was the most common comorbidity with 39.1% (*n* = 25). Chronic heart failure was present in 37.5% (*n* = 24) and 35.9% (*n* = 23) of the patients had diabetes. No patients with chronic obstructive pulmonary disease were observed in the alkalemia group (*n* = 0) compared with the acidemia (*n* = 8) and normal pH group (*n* = 1). Other comorbidities were relatively similar across groups, with no statistically significant differences in their frequency (Supplementary data, Table S1a).

**Table 2a:** Patient characteristics of the overall cohort.

	Overall
*N* (%)	64
Sex, female/male, *n* (%)	21/43 (32.8/67.2)
Age at ICU admission, years	68 (57–76)
Comorbidities	
Diabetes, *n* (%)	23 (35.9)
COPD, *n* (%)	9 (14.1)
Immunosuppression, *n* (%)	9 (14.1)
Chronic heart failure, *n* (%)	24 (37.5)
Liver disease, *n* (%)	18 (28.1)
Chronic kidney disease, *n* (%)	25 (39.1)
Long-term oxygen therapy, *n* (%)	3 (4.7)
BMI, kg/m^2^	25 (23–30)
SAPS 3, median (IQR)	76 (66–87)
Respiratory status	
Mechanical ventilation, *n* (%)	42 (65.6)

Data are presented as median (IQR) or *n* (%).

COPD, chronic obstructive pulmonary disease; BMI, body mass index.

**Table 2b:** Baseline laboratory parameters of the overall cohort.

	Overall
*N* (%)	64
Creatinine, mg/dL	2.8 (2.2–3.9)
Cystatin C, mg/L	3.5 (2.7–4.9)
pH	7.35 (7.27–7.40)
HCO_3_^–^, mmol/L	20 (17–22)
Base excess, mmol/L	–5.7 (–9.4 to –2.0)
pCO_2_, mmHg	36.2 (28–45.5)
Anion gap, mmol/L	15.0 (12.2–18.1)
Lactate, mg/dL	13.5 (8–22)
Sodium, mmol/L	139.5 (135–144.3)
Potassium, mmol/L	4.3 (3.9–4.8)
Calcium, mmol/L	1.08 (0.99–1.18)
Chloride, mmol/L	109 (105–115)
Osmolarity, mmol/kg	286 (279–299)
Urea, mg/dL	148 (103–202
Phosphate, mmol/L	1.73 (1.46–2.03)
Albumin, mg/dL	2426 (1872–2973)

Data are presented as median (IQR) or *n* (%).

HCO_3_^−^, bicarbonate.

The median Simplified Acute Physiology Score 3 (SAPS 3) at admission was 76 (IQR 66–87). The SAPS 3 was higher in patients with acidemia (acidemia: 79 vs alkalemia: 69 vs normal pH: 74), although this difference did not reach statistical significance.

In total, 65.6% required mechanical ventilation (*n* = 42), with more patients in the acidemia and normal pH group (acidemia: 67%; normal pH: 80%) than in the alkalemia group (25%).

### Laboratory parameters at start of RRT

All baseline laboratory parameters, except for acid–base parameters, were comparable among the three groups at the initiation of RRT (Supplementary data, Table S1b).

The median serum creatinine was 2.8 mg/dL (IQR 2.2–3.9) and the median cystatin C was 3.5 mg/L (IQR 2.7–4.9). There were no significant differences between the groups. The same was true for serum urea, lactate levels and the calculated anion gap as well as for sodium, potassium, chloride, calcium and phosphate (Supplementary data, Table S1b).

Serum bicarbonate was significantly lower in patients with acidemia [17.1 mmol/L (IQR 13.5–20.6), *P* < .001] compared with patients with alkalemia [22.3 mmol/L (IQR 20.1–26.9)] or normal pH [22.1 mmol/L (IQR 20–23.9)]. At the start of RRT, pCO_2_ tended to be lower in patients in the alkalemia group [28 mmHg (IQR 24–35)] compared with those with acidemia [37 mmHg (IQR 26–48)] or normal pH [36 mmHg (IQR 30–39)] but this did not reach statistical significance. The calculated anion gap and lactate levels were not significantly different between groups (Table 2b, Supplementary data, Table S1b).

### CVVH settings

CVVH machine settings were comparable across the three groups (Table 3). The median treatment time was significantly longer in patients with acidemia [234 h (IQR 113–368)] compared with patients with alkalemia [103 h (IQR 66–189)] and a normal pH [113 h (IQR 73–243], *P* = .041, Table 4]. Details on the adjustments to CVVH settings after 48 h are available in Table 3. No significant differences were observed. Blood flow was slightly reduced in patients with alkalemia after 48 h (0 h: 130 mL/min; 48 h: 125 mL/min).

**Table 3:** CVVH settings at initiation and after 48 h for patients stratified by pH at start of RRT.

	Overall		Acidemia		Alkalemia		Normal pH	
Timepoint	0 h	48 h	0 h	48 h	0 h	48 h	0 h	48 h
Pre-dilution pre-blood pump fluid (Prismocitrate™), mL/h	1200 (1200.00–1300)	1200 (1200–1300)	1200 (1200–1225)	1200 (1200–1300)	1300 (1200–1300)	1250 (1200–1300)	1200 (1200–1200)	1200 (1200–1285)
Substitution rate, mL/h	800 (800–800)	800 (800–1000)	800 (800–1000)	800 (800–1000)	800 (775–800)	800 (800–850)	800 (700–800)	800 (700–800)
Fluid removal, mL/h	–50.00 (–110 to 0)	–100 (–200 to –100)	–50 (–150 to 0)	–100 (–200 to –100)	0 (0–0)	–100 (–138 to –75)	–100 (–100 to 0)	–100 (–175 to –88)
Blood flow, mL/min	120 (120–130)	120 (120–130)	120 (120–130)	120 (120–130)	130 (125–130)	125 (120–130)	120 (120–120)	120 (120–123)
Citrate dose, mmol/L	3 (3–3)	3 (3–3)	3 (3–3)	3 (3–3)	3 (3–3)	3 (3–3)	3 (3–3)	3 (3–3)
Calcium dose, mmol/h	1.7 (1.5–2)	2.8 (2.3–3.2)	1.7 (1.5–2)	2.8 (2.3–2.3)	1.8 (1.7–2)	2.4 (1.8–3.2)	1.8 (1.6–2)	2.7 (2.3–3)
Calcium substitution, %	78 (70–80)	105 (90–110)	70 (70–80)	105 (90–115)	75 (70–80)	95 (78–115)	80 (70–81)	103 (94–105)

Data are presented as median (IQR).

**Table 4:** CVVH duration, length of stay and mortality of patients stratified by pH at start of RRT.

	Overall	Acidemia	Alkalemia	Normal pH	*P*
CVVH duration	177 (74–344)	234 (113–368)	103 (66–189)	113 (73–243)	*.041*
ICU LOS, days	15 (9–24)	15 (8–23)	10 (8–20)	15 (11–26)	.504
Hospital LOS, days	29 (18–43)	32 (18–45)	13 (11–33)	29 (27–41)	.259
ICU mortality, *n* (%)	27 (42.2)	14 (38.9)	5 (62.5)	8 (40)	.460
Hospital mortality, *n* (%)	36 (56.2)	18 (50)	6 (75)	12 (60)	.401

Data are presented as median (IQR) or *n* (%).

LOS, length of stay.

### Course of metabolic parameters during RRT

**Figure 1:**
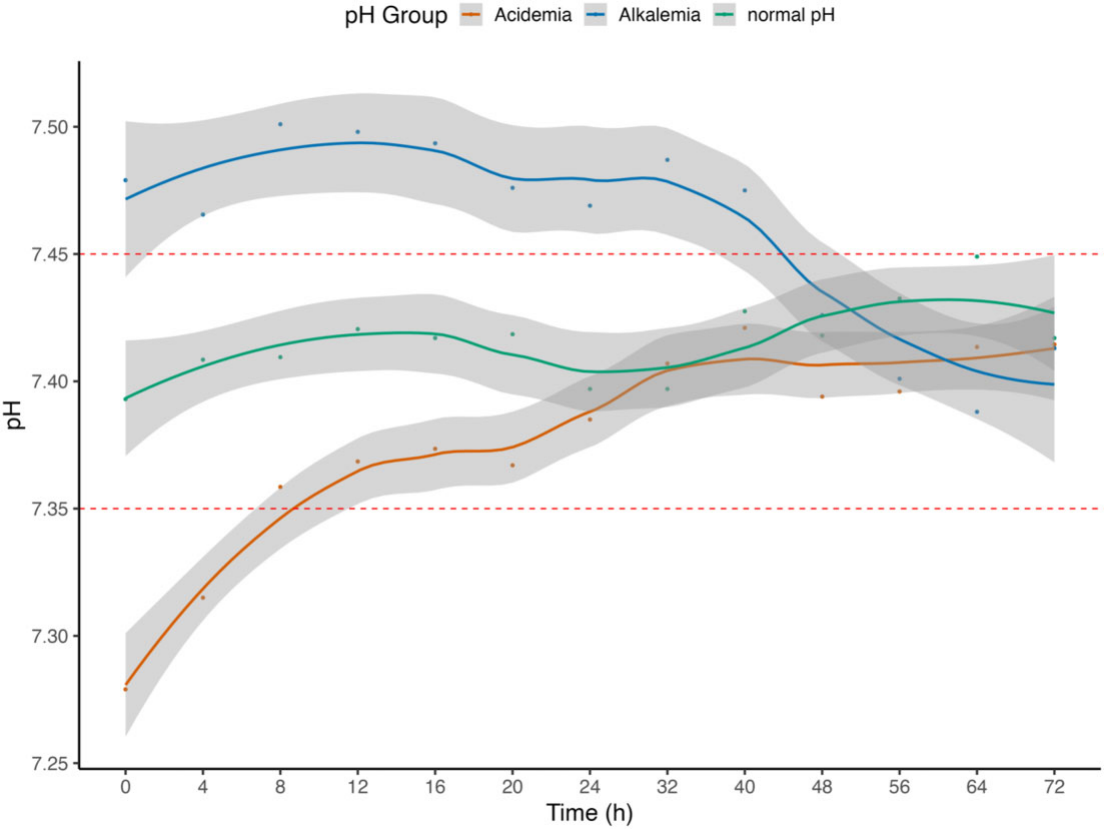
Course of pH in CVVH patients with RCA and a high bicarbonate RF over time (h) stratified by pH at start of RRT.

**Figure 2:**
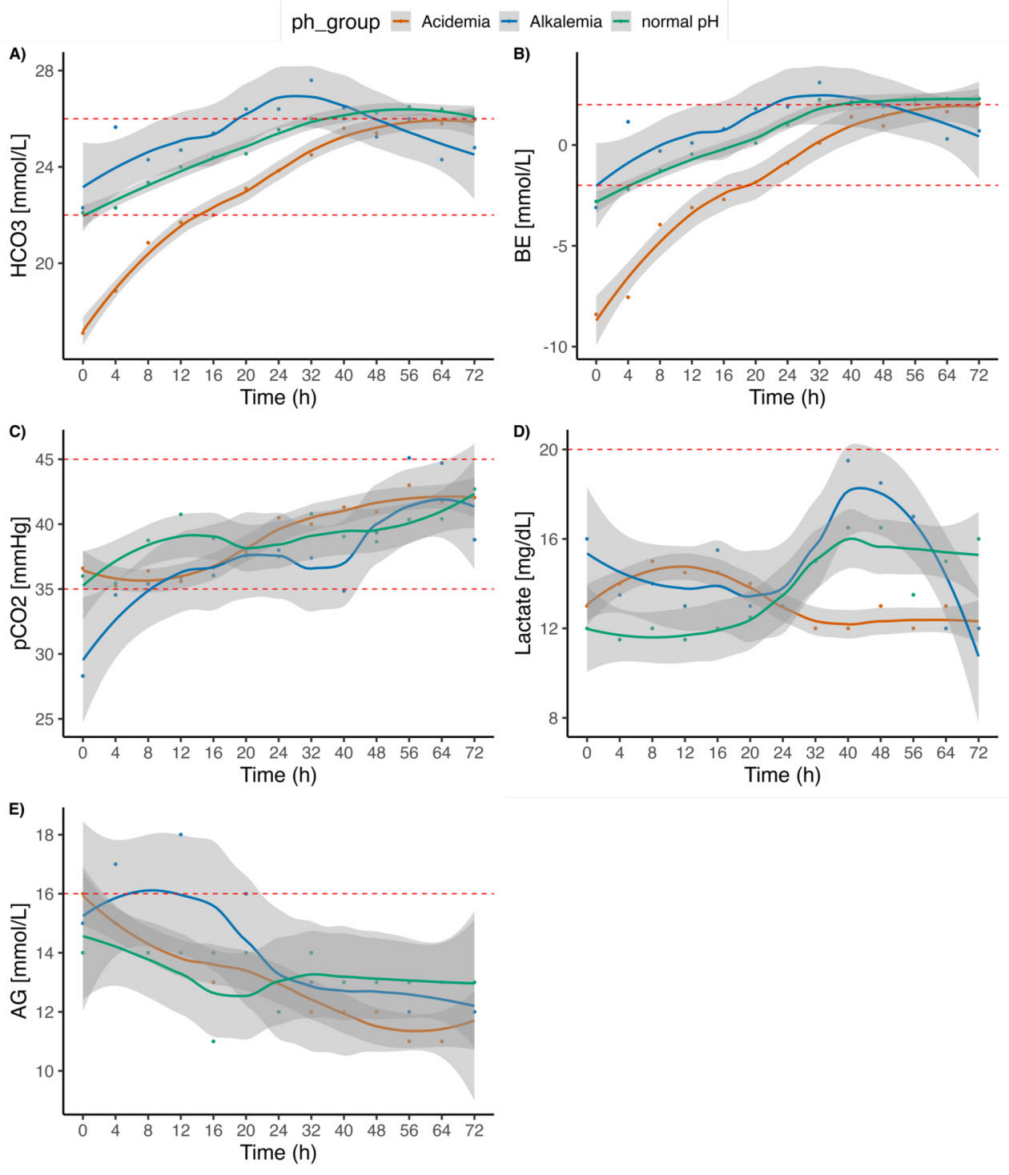
Course of bicarbonate (HCO_3_^–^) (**A**), base excess (**B**), pCO_2_ (**C**), lactate (**D**) and anion gap (**E**) in CVVH patients with RCA and a high bicarbonate RF over time (h) stratified by pH at start of RRT.

At the initiation of CRRT most patients had an acidemia with 36 (56.3%), compared with 8 (12.5%) patients with an alkalemia and 20 (32.3%) patients with a normal pH (Supplementary data, Table S1b). Figures 1 and 2A–E illustrate the course of acid–base parameters following the initiation of RRT over time. While the median pH of patients with an acidemia at the beginning of treatment reached and stayed within a normal range after 8 h, a normal median pH was reached and sustained after 48 h in the alkalemia group after start of RRT (see Fig. 1, red and green line, Supplementary data, Table S2). Patients with a normal pH (Fig. 1, blue line) experienced a further slight increase of pH over 72 h but remained within the normal range. The bicarbonate levels and the base excess increased in all patients (Supplementary data, Fig. S2), with the greatest increase in patients with acidemia (Fig. 2A and B). Conversely the chloride and sodium levels decreased in all patients over time (Supplementary data, Fig. S2). The SID over time is shown in Supplementary data, Fig. S3 and showed a trend towards higher values over time.

During the period from 24 to 72 h, all groups showed a steady increase in pCO_2_ levels (Fig. 2C, Supplementary data, Fig. S2). Conversely, the calculated anion gap (including potassium) showed a decrease over the observed period (Fig. 2E). Lactate levels varied considerably across all groups (Fig. 2D).

### Mortality

Median ICU and hospital length of stay was longer in patients with acidemia (ICU: 15 days; hospital: 32 days) and normal pH (ICU: 15 days; hospital: 29 days) compared with patients with alkalemia (ICU: 10 days; hospital: 13 days).

Overall hospital mortality was 56.2% (*n* = 36). Hospital mortality was higher in patients with alkalemia (75%, *n* = 6) compared with patients with acidemia (50%, *n* = 18) or a normal pH (60%, *n* = 12), but the differences were not statistically significant.

## DISCUSSION

Acid–base disturbances are common in critically ill patients undergoing CRRT, yet evidence on the composition of RFs and their impact on acid–base balance remains limited. In our cohort, median pH normalized quickly after 8 h for patients with acidemia. Over 72 h, all patients showed a trend towards elevated bicarbonate levels and base excess. Our results reveal different dynamics in the rate of correction of acid–base disturbances and underscore the importance of the choice of RF in CVVH for critically ill patients.

Continuous delivery of RRT has become the standard-of-care in ICUs, as recommended by the KDIGO guidelines [5]. When using CVVH, a RF is required to substitute for intracorporeal losses (fluid, electrolytes etc.) due to the extracted ultrafiltrate. Initially, when the use of continuous methods for RRT became more common, there were no specific RFs available for critically ill patients and their individual needs. As a result, early studies on RFs focused on their electrolyte composition, e.g. phosphate [16–18]. All RFs used in these studies contain relatively high bicarbonate concentrations as they were developed for heparin anticoagulation during CRRT. With the introduction of RCA and its benefits, such as an extended filter-life (9), high bicarbonate concentrations in these RFs may no longer be necessary, as citrate is metabolized to bicarbonate mainly in the liver.

### Time to normalization in patients with acidemia

In our study, most patients presented with acidemia at the initiation of CVVH, which is consistent with large interventional RRT trials [19, 20]. Correction of acidemia occurred rapidly, after 8 h (7.36, IQR 7.29–7.4) the median pH reached the normal range. The mechanism behind this fast correction is likely multifactorial, involving the direct buffering effect of bicarbonate and the removal of acidotic components by CRRT in general. Few studies have investigated the complex influence of CRRT on acid–base balance. In 2003, Rocktäschel *et al*. demonstrated acidemia correction during CVVH after 24 h [21]. In their study, heparin anticoagulation and lactate buffered RFs were used. Therefore, the use of a high bicarbonate concentration RF together with the use of RCA in this study may be responsible for the observed difference in the time to normalization of the pH in acidotic patients. However, it remains unclear whether this faster correction would translate into improved outcomes.

Only a small fraction of our patients presented with alkalemia at the initiation of CRRT. Notably, the combination of normal bicarbonate levels and reduced pCO_2_ suggests a predominantly respiratory origin for the alkalosis in these cases. As expected, pH correction was delayed in this group (48 h). Our mortality analysis showed a trend toward higher, albeit not statistically significant in patients with alkalosis compared with patients with acidosis or normal pH. It remains uncertain whether the additional bicarbonate load from the RF exacerbates metabolic alkalosis, potentially impairing oxygen delivery and cellular function. However, the small sample size and the lack of respiratory data limit a more definitive conclusion about the role of the RF in these patients.

### Steady-state conditions

Interestingly, all patients showed a continuous upward trend even after normalization over the 72-h period towards a metabolic alkalosis with elevated bicarbonate and base excess compared with the baseline. However, this was offset by an increase on pCO_2_ and, thus, the majority of patients maintained their pH within the normal range.

Some citrate solutions have a relatively high sodium concentration and can contribute to plasma alkalinization by increasing the SID. However, unlike high-sodium citrate solutions, we used Regiocit^®^ (Prismocitrate 18/0), which contains 140 mmol/L of sodium, minimizing this effect. This is supported by our findings that sodium levels decreased over time in our cohort (Supplementary data, Fig. S2). Despite this, SID showed a slight increase over time, which may be explained by the progressive decline in chloride levels (Supplementary data, Fig. S2). Interestingly, this was seen despite the use of a chloride-rich solution such as Phoxilium^®^ used for post-filter substitution. We believe that this can be explained by several factors. First, Regiocit^®^ administered in predilution, contains only 86 mmol/L of chloride and was infused at a higher rate than the post-filter substitution fluid Phoxilium^®^, which may have contributed to a relative chloride depletion. Second, chloride clearance may have been enhanced due to the Gibbs–Donnan effect, as has also been shown for CVVH in a recent study [22].

Finally, solute clearance during CVVH occurs from the plasma water compartment, where the effective chloride concentration is higher than the measured plasma concentration due to plasma being approximately 93% water. This may result in greater chloride clearance than anticipated.

Our findings are similar to the study by Rocktäschel *et al*. describing that CVVH corrects acidemia. However, their study reported alkalemia at 72 h [21], which could be attributed to the use of a lactate buffer with a concentration of 46 mmol/L and heparin anticoagulation, in contrast to our study, which used a 30 mmol/L bicarbonate solution with RCA. We cannot rule out that patients developed metabolic alkalemia after the study period of 72 h. In addition, while patients might develop an alkalemia, its impact on outcomes remains unclear. An observational study from 2008 of 405 patients on bicarbonate based continuous haemodialysis, found no association between alkalemia and increased mortality [23]. However, in 2017 Kashani *et al*. [8] suggested a potential negative impact on mortality with higher bicarbonate solutions (32 mmol/L vs 22 mmol/L) during CVVH with RCA.

These studies highlight the importance of RF composition and reinforce the findings of our study. The occurrence of alkalemia appears to be a common issue during CVVH, and switching to the currently available low-bicarbonate RFs has been shown to reduce bicarbonate levels [15]. It remains to be determined whether, in addition to other possible interventions like reducing the citrate dose or the blood flow, the use of different solutions would improve acid–base control and clinical outcomes.

### Strengths and limitations

The main strengths of this study are the availability of longitudinal acid–base parameters over a period of 72 h, the comparability of the study population due to the use of clear inclusion and exclusion criteria and a standardized CVVH with RCA treatment protocol in all patients. The consistent CVVH settings across groups and over time further reinforce the robustness of our findings, minimizing the potential confounding effects of varying treatment protocols. Furthermore, this is not only the first study to investigate the influence of high bicarbonate RFs on acid–base balance after initiation of RRT, but also one of the the largest studies on this subject.

Citrate accumulation, though rare, remains an important consideration in citrate-based therapies [23]. In our cohort, calcium infusion rates remained stable throughout treatment, and no clinical or biochemical markers indicative of citrate accumulation were observed. While we did not detect citrate accumulation during the 72-h study period, we cannot exclude the possibility that it may have occurred later during prolonged CVVH. The main limitations arise from the observational, retrospective design of our study. Because some important variables, such as ventilator settings, were not available, we could not consider the possible associated effects on acid–base balance. In addition, the sample size, especially in patients with alkalemia, does not allow for definitive conclusions regarding clinical outcomes.

## CONCLUSION

The choice of RF during CVVH with RCA appears to be one of several factors influencing acid–base status in critically ill patients. Our findings suggest that patients with different acid–base disorders at the start of CRRT exhibit distinct correction kinetics when treated with high bicarbonate RF. Whether the choice of RF translates into better clinical outcomes remains unclear. Further prospective studies are needed to directly compare different RF compositions and better understand their impact on acid–base balance and patient outcomes.

## Supplementary Material

sfaf117_Supplemental_File

## Data Availability

The datasets used and analyzed during the current study are available from the corresponding author on reasonable request.

## References

[1] Hoste EAJ, Bagshaw SM, Bellomo R et al. Epidemiology of acute kidney injury in critically ill patients: the multinational AKI-EPI study. Intensive Care Med 2015;41:1411–23. 10.1007/s00134-015-3934-7 26162677

[2] Rabindranath KS, Adams J, MacLeod AM et al. Intermittent versus continuous renal replacement therapy for acute renal failure in adults. Cochrane Database Syst Rev 2007:CD003773. 17636735 10.1002/14651858.CD003773.pub3 PMC13199906

[3] Klein SJ, Joannidis M. Nierenersatztherapie im akuten Nierenversagen. Intensivmed Notfallmed 2017;112:437–43. 10.1007/s00063-017-0290-0 28466293

[4] Tolwani A. Continuous renal-replacement therapy for acute kidney injury. N Engl J Med 2012;367:2505–14. 10.1056/NEJMct1206045 23268665

[5] Legrand M, Darmon M, Joannidis M et al. Management of renal replacement therapy in ICU patients: an international survey. Intensive Care Med 2013;39:101–8. 10.1007/s00134-012-2706-x 23001448

[6] Jaber S, Paugam C, Futier E et al. Sodium bicarbonate therapy for patients with severe metabolic acidaemia in the intensive care unit (BICAR-ICU): a multicentre, open-label, randomised controlled, phase 3 trial. Lancet 2018;392:31–40. 10.1016/S0140-6736(18)31080-8 29910040

[7] Kraut JA, Madias NE. Sodium bicarbonate for severe metabolic acidaemia. Lancet 2018;392:3–4. 10.1016/S0140-6736(18)31305-9 29910039

[8] Kashani K, Thongprayoon C, Cheungpasitporn W et al. Association between mortality and replacement solution bicarbonate concentration in continuous renal replacement therapy: a propensity-matched cohort study. PLoS One 2017;12:e0185064. 10.1371/journal.pone.0185064 28957333 PMC5619733

[9] Khwaja A. KDIGO clinical practice guidelines for acute kidney injury. Nephron Clin Pract 2012;120:c179–84. 10.1159/000339789 22890468

[10] Oudemans-van Straaten HM, Ostermann M. Bench-to-bedside review: citrate for continuous renal replacement therapy, from science to practice. Crit Care 2012;16:249. 10.1186/cc11645 23216871 PMC3672558

[11] Schneider AG, Journois D, Rimmelé T. Complications of regional citrate anticoagulation: accumulation or overload? Crit Care 2017;21:281. 10.1186/s13054-017-1880-1 29151020 PMC5694623

[12] Gunnerson KJ, Saul M, He S et al. Lactate versus non-lactate metabolic acidosis: a retrospective outcome evaluation of critically ill patients. Crit Care 2006;10:R22. 10.1186/cc3987 16507145 PMC1550830

[13] Yessayan L, Yee J, Frinak S et al. Continuous renal replacement therapy for the management of acid-base and electrolyte imbalances in acute kidney injury. Adv Chronic Kidney Dis 2016;23:203–10. 10.1053/j.ackd.2016.02.005 27113697

[14] Yessayan L, Yee J, Frinak S et al. Treatment of severe metabolic alkalosis with continuous renal Replacement therapy: bicarbonate kinetic equations of clinical value. ASAIO J 2015;61:e20–5. 10.1097/MAT.0000000000000216 25794247

[15] Köglberger P, Klein SJ, Lehner GF et al. Low bicarbonate replacement fluid normalizes metabolic alkalosis during continuous veno-venous hemofiltration with regional citrate anticoagulation. Ann Intensive Care 2021;11:62. 10.1186/s13613-021-00850-4 33891213 PMC8062940

[16] Chua H-R, Baldwin I, Ho L et al. Biochemical effects of phosphate-containing replacement fluid for continuous venovenous hemofiltration. Blood Purif 2012;34:306–12. 10.1159/000345343 23235269

[17] Chua H-R, Schneider AG, Baldwin I et al. Phoxilium vs hemosol-B0 for continuous renal replacement therapy in acute kidney injury. J Crit Care 2013;28:884.e7–14. 10.1016/j.jcrc.2013.02.013 23683569

[18] Besnard N, Serveaux M, Machado S et al. Electrolytes-enriched hemodiafiltration solutions for continuous renal replacement therapy in acute kidney injury: a crossover study. Blood Purif 2016;42:18–26. 10.1159/000444248 26949936

[19] Gaudry S, Hajage D, Martin-Lefevre L et al. Comparison of two delayed strategies for renal replacement therapy initiation for severe acute kidney injury (AKIKI 2): a multicentre, open-label, randomised, controlled trial. Lancet 2021;397:1293–300. 10.1016/S0140-6736(21)00350-0 33812488

[20] STARRT-AKI Investigators; Canadian Critical Care Trials Group; Australian and New Zealand Intensive Care Society Clinical Trials Group; United Kingdom Critical Care Research Group; Canadian Nephrology Trials Network; Irish Critical Care Trials Group; Bagshaw SM, Wald R, Adhikari NKJ et al. Timing of initiation of renal-replacement therapy in acute kidney injury. N Engl J Med 2020;383:240–51. 10.1056/NEJMoa2000741 32668114

[21] Rocktäschel J, Morimatsu H, Uchino S et al. Impact of continuous veno-venous hemofiltration on acid-base balance. Int J Artif Organs 2003;26:19–25. 10.1177/039139880302600104 12602465

[22] Zadek F, Brunoni B, Mulazzani F et al. Acid–base implications of the Gibbs-Donnan effect during continuous veno-venous hemofiltration. J Nephrol 2025. Epub ahead of print. 10.1007/s40620-025-02238-0 PMC12166026 40059273

[23] Demirjian S, Teo BW, Paganini EP. Alkalemia during continuous renal replacement therapy and mortality in critically ill patients. Crit Care Med 2008;36:1513–7. 10.1097/CCM.0b013e318170a2f5 18434895

