# Supplementary material for: High bicarbonate replacement fluid and time to pH normalization during continuous veno-venous hemofiltration with regional citrate anticoagulation: a retrospective single-center cohort study
Source: Clin Kidney J. 2025 Apr 26;18(5):sfaf117. doi: 10.1093/ckj/sfaf117 (PMC12067065; doi:10.1093/ckj/sfaf117)

High bicarbonate replacement fluid and time to pH normalization during Continuous Venovenous Hemofiltration with Regional Citrate Anticoagulation– a retrospective single-center cohort study

Tables

Supplemental Table 1a: Patient characteristics stratified by pH at start of RRT

	Overall	Acidemia	Alkalemia	normal pH	*p*
**N (%)**	64	36 (56.3)	8 (12.5)	20 (32.3)	
**Sex, female (%)**	21 (32.8)	12 (33.3)	2 (25.0)	8 (35)	0.874
**Age at ICU admission, years**	68 [57 - 76]	68 [60 - 75]	73 [60 - 77]	68 [55 - 75]	0.774
Comorbidities					
**Diabetes, n (%)**	23 (35.9)	15 (41.7)	3 (37.5)	5 (25)	0.458
**COPD, n (%)**	9 (14.1)	8 (22.2)	0 (0)	1 (5)	0.098
**Immunosuppression, n (%)**	9 (14.1)	12 (33.3)	2 (25.0)	5 (25)	0.770
**Chronic heart failure, n (%)**	24 (37.5)	13 (36.1)	4 (50.0)	7 (35)	0.735
**Liver disease, n (%)**	18 (28.1)	9 (25)	3 (37.5)	6 (30)	0.757
**Chronic kidney disease, n (%)**	25 (39.1)	15 (41.7)	3 (37.5)	7 (35)	0.883
**Long term oxygen therapy, n (%)**	3 (4.7)	3 (8.3)	0 (0.0)	0 (0.0)	0.294
**BMI, kg/m^2**	25 [23 - 30]	27 [23 - 30]	25 [23 - 27]	25 [24 - 30]	0.727
**SAPS 3**	76 [66 - 87]	79 [68 - 89]	69 [61 - 73]	74 [66 - 87]	0.092
Respiratory status					
**Mechanical ventilation, n (%)**	42 (65.6)	24 (67)	2 (25)	16 (80)	0.021

ICU – intensive care unit, COPD - chronic obstructive pulmonary disease, BMI – body mass index, SAPS - Simplified Acute Physiology Score,

Supplemental Table 1b: Baseline laboratory parameters stratified by pH at start of RRT

	Overall	Acidemia	Alkalemia	normal pH	*p*
**Creatinine, mg/dl**	2.8 [2.2 - 3.9]	2.9 [2.3 - 3.7]	3 [1.9 - 4]	2.6 [2.4 – 4.2]	0.947
**Cystatin C, mg/l**	3.5 [2.7 - 4.9]	3.4 [2.6 - 4.2]	3.7 [3 - 5]	3.9 [2.9 - 5]	0.396
**pH**	7.35 [7.27 - 7.40]	7.28 [7.23 - 7.33]	7.49 [7.47 - 7.51]	7.39 [7.38 - 7.41]	<0.001
**HCO3-, mmol/l**	19 [16.8 - 22.3]	17.1 [13.5 - 20.6]	22.3 [20.1 - 26.9]	22.1 [20 – 23.9]	<0.001
**Base excess**	-5.7 [-9.4 - -2]	-8.4 [-14, -4.5]	-3.1 [-6 - 2.5]	-2.8 [-5.9 - 0.7]	0.002
**pCO2, mmHg**	36 [28 – 46]	37 [26 - 48]	28 [24 - 35]	36 [30 - 39]	0.271
**Anion gap, mmol/l**	15 [12.2 - 18.1]	16.1 [12.4 - 18.4]	15.3 [14.6 - 17.4]	14.1 [11.5 - 17.4]	0.479
**Lactate, mg/dl**	13.5 [8 - 22]	13 [7.5 - 25.5]	16 [13.8 - 19.8]	12 [8 – 19.5]	0.519
**Sodium, mmol/l**	140 [135 - 144]	138 [135 - 143]	147 [140 - 154]	140 [135 - 145]	0.268
**Potassium, mmol/l**	4.3 [3.9 - 4.8]	4.5 [3.9 - 5.3]	4.2 [3.9 - 4.9]	4.1 [3.7 - 4.3]	0.094
**Calcium, mmol/l**	1.08 [0.99 - 1.18	1.10 [1.02 - 1.19]	1.13 [1.05 - 1.22]	1.04 [0.98 - 1.17]	0.308
**Chloride, mmol/l**	109 [105 – 115]	110 [106 - 116]	111 [108 - 117]	106 [105 - 112]	0.340
**Magnesium, mmol/l**	0.91[0.77-1.09]	0.91 [0.76-1.03]	0.98 [0.59-1.10]	0.91 [0.81-1.16]	0.860
**Osmolarity,**	286 [279 - 299]	284 [279 - 294]	303 [290 - 316]	287 [278 - 298]	0.219
**Urea, mg/dl**	148 [103 - 202]	147 [90 - 205]	158 [138 - 193]	146 [107 - 194]	0.667
**Phosphate, mmol/l**	1.73 [1.46 - 2.03]	1.83 [1.54 - 2.34]	1.56 [1.15 - 1.78]	1.62 [1.44 - 1.82]	0.089
**Albumin, mg/dl**	2426 [1872 -2973]	2451 [2145 – 3205]	2909 [2572 - 2971]	1994 [1723 - 2132]	0.128

Supplemental Table 2: Median pH at selected timepoints for patients stratified by pH at start of RRT

	0h	8h	16h	24h	48h	72h
**Acidemia**	7.28 (7.23 - 7.33)	**7.36 (7.29 - 7.4)**	7.37 (7.32 - 7.45)	7.38 (7.33 - 7.43)	7.39 (7.36 - 7.44)	7.41 (7.37 - 7.45)
**Alkalemia**	7.48 (7.47- 7.49)	7.5 (7.45 - 7.52)	7.49 (7.46 - 7.51)	7.47 (7.43 - 7.48)	**7.43 (7.39 - 7.46)**	7.41 (7.39 - 7.44)
**Normal pH**	7.39 (7.38 - 7.41)	7.41 (7.37 - 7.44)	7.42 (7.39 - 7.46)	7.4 (7.37 - 7.45)	7.42 (7.4 - 7.45)	7.42 (7.38 - 7.47)

Figures

Supplemental Figure 1: Flowchart of excluded and included patients

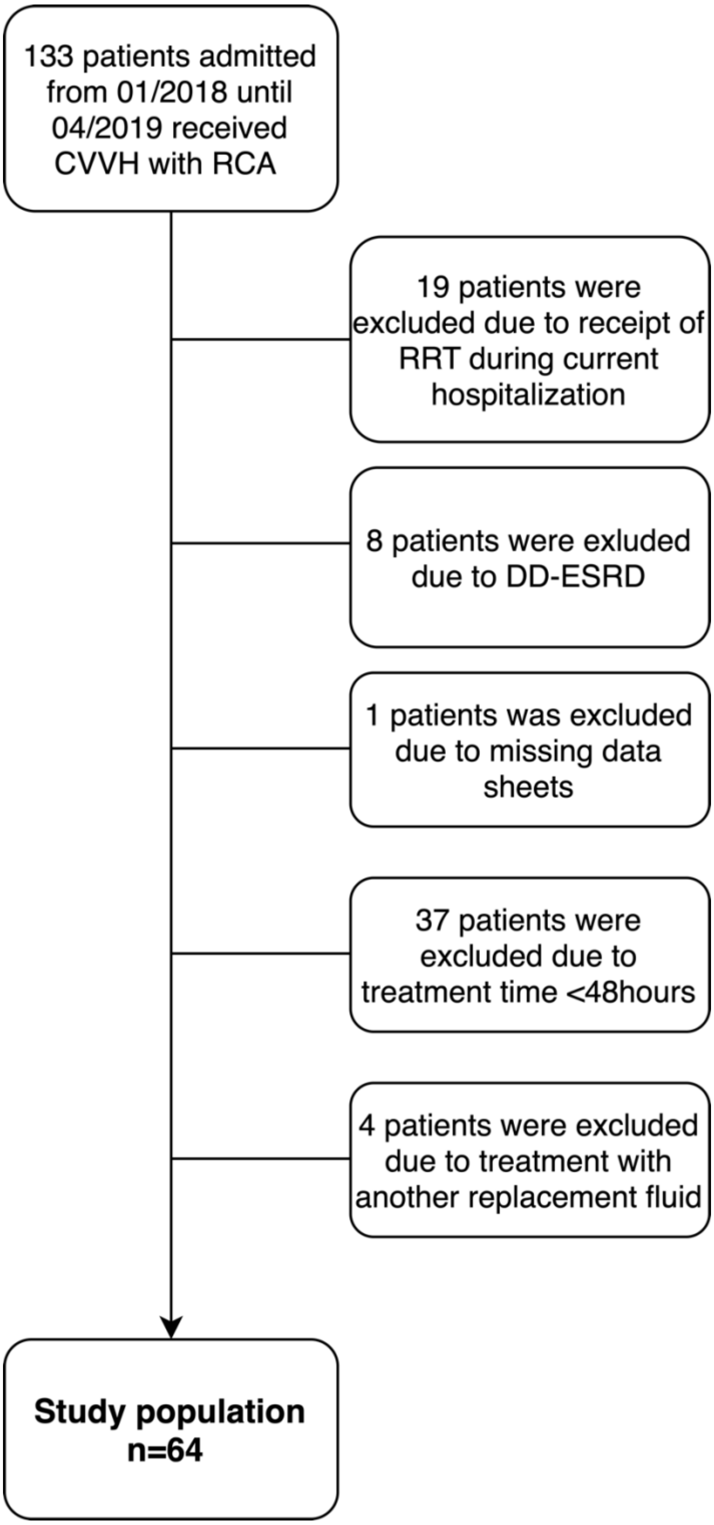

Supplemental Figure 2: Changes of pH, HCO3-, base excess (BE), pCO2, lactate, anion gap (AG), chloride (Cl) and sodium (Na) in all patients at 0, 24, 48 and 72 hours

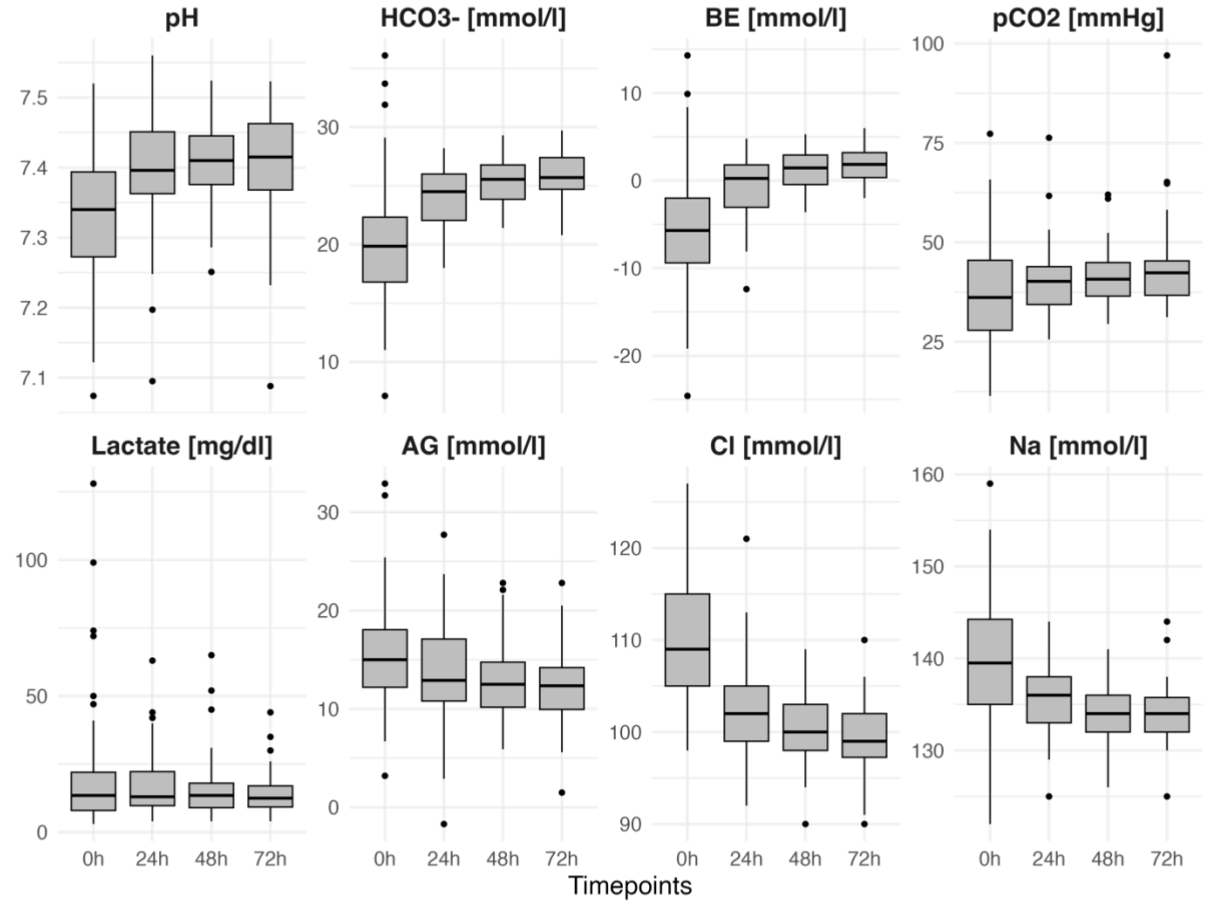

Supplemental Figure 3: Changes of the strong ion difference (SID) in all patients at 0, 24, 48 and 72 hours

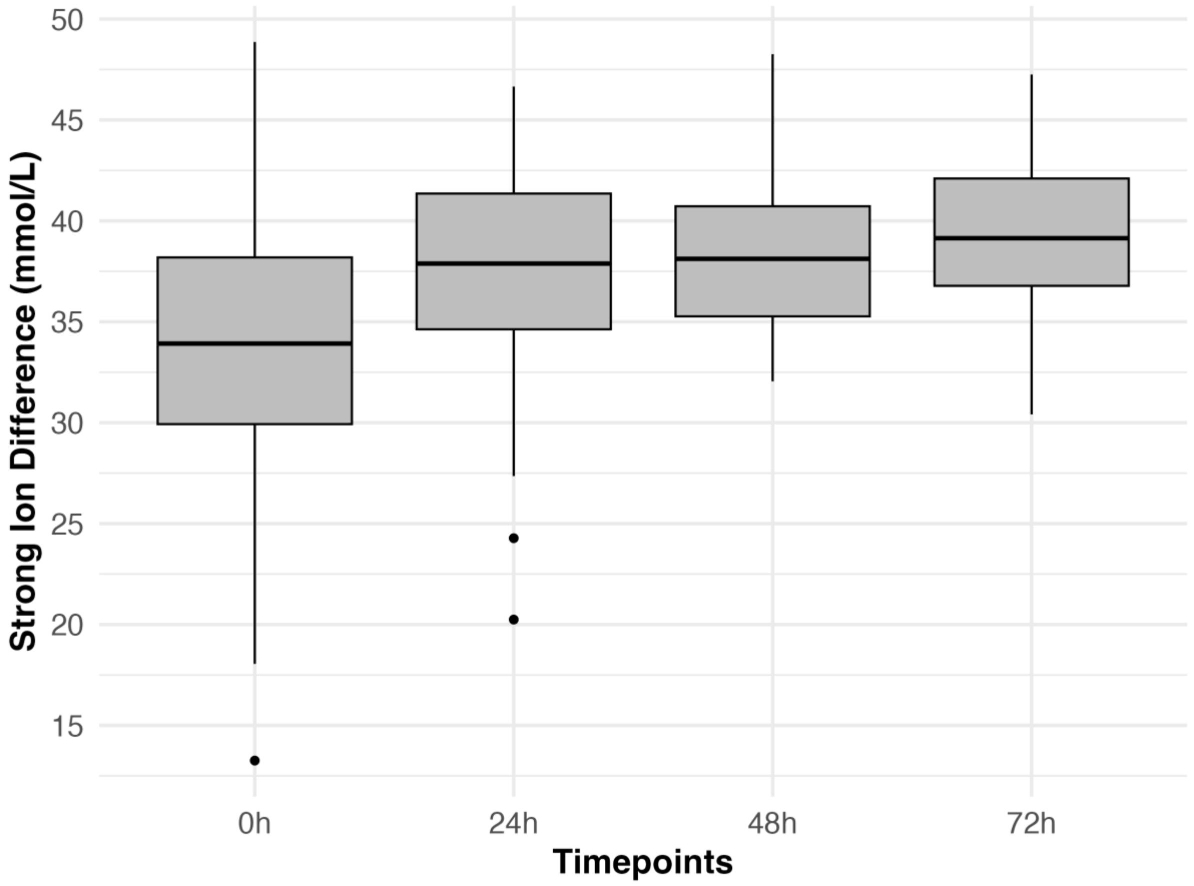

Supplement: sfaf117_Supplemental_File [file sfaf117_supplemental_file.pdf]
